# Effect of Passive Warm-Up Using High-Voltage Pulsed-Current Electrical Stimulation on Jump Performance in Young Adults: A Randomized Controlled Trial

**DOI:** 10.7759/cureus.81594

**Published:** 2025-04-02

**Authors:** Michio Wachi, Takumi Jiroumaru, Ayako Satonaka, Katsuyoshi Tanaka, Junko Ochi, Yutaro Hyodo, Nobuko Shichiri, Takamitsu Fujikawa

**Affiliations:** 1 Department of Physical Therapy, Bukkyo University, Kyoto, JPN; 2 Department of Applied Biology, Aichi Shukutoku University, Aichi, JPN; 3 Department of Occupational Therapy, Bukkyo University, Kyoto, JPN

**Keywords:** core training, electrical stimulation, high-voltage pulsed current, jump performance, passive warm-up

## Abstract

Background

Warm-ups optimize athletic performance; however, excessively active warm-ups may induce fatigue. Passive warm-up strategies, including thermotherapy, offer physiological benefits, although their immediate performance effects are unclear. High-voltage pulsed-current (HVPC) electrical stimulation, used in rehabilitation to activate deep muscles, has not been fully explored as a warm-up strategy. This study examined the immediate effects of short-duration HVPC stimulation combined with abdominal hollowing exercises on jump performance.

Methods

A total of 36 healthy participants were randomly assigned to the HVPC (n = 18) or control (n = 18) group. The HVPC group performed abdominal hollowing exercises with HVPC stimulation for 4 minutes, while the control group performed the exercises without electrical stimulation. The rebound jump (RJ) index, jump height, and ground contact time were measured before and after the intervention. A paired t-test was used to compare the pre- and post-experiment measurements.

Results

The HVPC group showed a significant increase in jump height (pre 31.95 ± 6.42 vs post 33.64 ± 6.61) (*p* < 0.05) and RJ index (pre 1.67 ± 0.47 vs post 1.75 ± 0.44) (*p* < 0.05) post-intervention, whereas the ground contact time remained unchanged. The control group showed no significant changes in any parameter.

Conclusion

Short-duration HVPC stimulation combined with abdominal hollowing exercises improved jump performance. HVPC-assisted warm-ups show potential, particularly in sports.

## Introduction

Warm-ups before exercise aim for optimal performance and are extensively practiced by athletes and recreational sports participants. Additionally, a recent review reported that warm-ups can reduce sports injuries by 36%, highlighting their usefulness in injury prevention [1]. According to Neiva et al., the ideal warm-up should occur within 30 minutes before a match [2]. Unlike regular training, warm-ups must be brief yet effective. Although the optimal warm-up method is undetermined, evidence supports the benefits of various approaches [3-6]. A review by McGowan et al. found that warm-ups enhance body temperature, muscle metabolism, fiber conduction velocity, and psychological readiness [4]. An essential aspect of warm-up is employing strategies that efficiently elicit these physiological responses. Warm-up methods are broadly classified as active (direct physical activity) and passive (indirect external stimuli), each with distinct effects. However, while active warm-ups can improve performance, excessive exertion may lead to fatigue and diminished performance [7].

We previously examined a passive warm-up strategy that enhanced performance without energy consumption. The application of a thermotherapy device to trunk muscles immediately improved muscle activation timing, muscle stiffness, and the active straight leg raise angle [8,9]. These effects persisted for at least 30 minutes. The physiological effects of thermotherapy likely stem from increased muscle temperature, which enhances adenosine triphosphate turnover, crossbridge cycling, muscle fiber function, and conduction velocity [10]. Conversely, while increased muscle temperature enhances certain physiological properties, it does not significantly reduce reaction time or alter coordinated movement and postural control [11]. Another study also investigated passive warm-up using electrical or heat therapy devices; however, it only focused on temperature, which was insufficient to effectively enhance performance [12].

Electrical muscle stimulation efficiently activates muscles and enhances contraction, making it valuable in clinical settings for strengthening muscles, neuromuscular reeducation, motor control, and maintaining muscle function during prolonged immobilization [13-15]. High-voltage pulsed-current (HVPC) electrical stimulation can reach the deep muscles while also stimulating sensory and motor nerve fibers. Owing to its short pulse duration (≤100 µs), it avoids stimulating pain-sensitive Aδ and C nerve fibers by minimizing discomfort [16]. Several studies have reported that electrical stimulation activates the deep trunk muscles [14,17]. Cho et al. used ultrasonography to assess muscle thickness during lateral abdominal electrical stimulation and confirmed contraction of the transverse abdominis muscle [14], which is located deep within the trunk, and it is therefore difficult to monitor and visualize. Even in healthy individuals, activating the transverse abdominis muscle is challenging, and muscle activity increases when assisted by physical therapy equipment [18]. However, previous studies have not clarified the immediate effects of electrical muscle stimulation on performance. Additionally, previous studies applied electrical muscle stimulation for 20 minutes per session [13-15], which may be impractical for warm-up routines.

Based on these findings, this study examined the immediate effects of short-duration HVPC stimulation on trunk muscle performance.

## Materials and methods

Participants

A total of 36 healthy young male and female individuals who did not exercise regularly (at least four days a week) were recruited from two universities in Japan from December 2024 to January 2025. The participants had no history of lower back pain, previous abdominal or lower back surgery, infection, neurological or sciatic nerve root compression, sensory impairment, or decreased muscle strength. With an effect size of d = 0.8, a significance level of 5%, and a power of 80%, the required sample size for the paired t-test was 15 participants per group. Twenty participants were recruited per group to account for potential dropouts or incomplete data. The sample size was calculated using G*Power 3.1 [19]. The participants were randomly categorized into either the HVPC group (n = 18) or the control group (n = 18) using a computer-generated randomization sequence. The participants and statisticians were blinded to participant allocation. The study protocol was approved by the Ethics Committee of the Kanazawa Orthopaedic Sports Medicine Clinic, Kanazawa, Japan (OSMC-2024-011). Written informed consent was obtained from all the participants. All study procedures adhered to the standards of the Declaration of Helsinki. This clinical study was registered with the University Hospital Medical Information Network in Japan (UMIN000056416).

Experimental procedure

**Figure 1:**
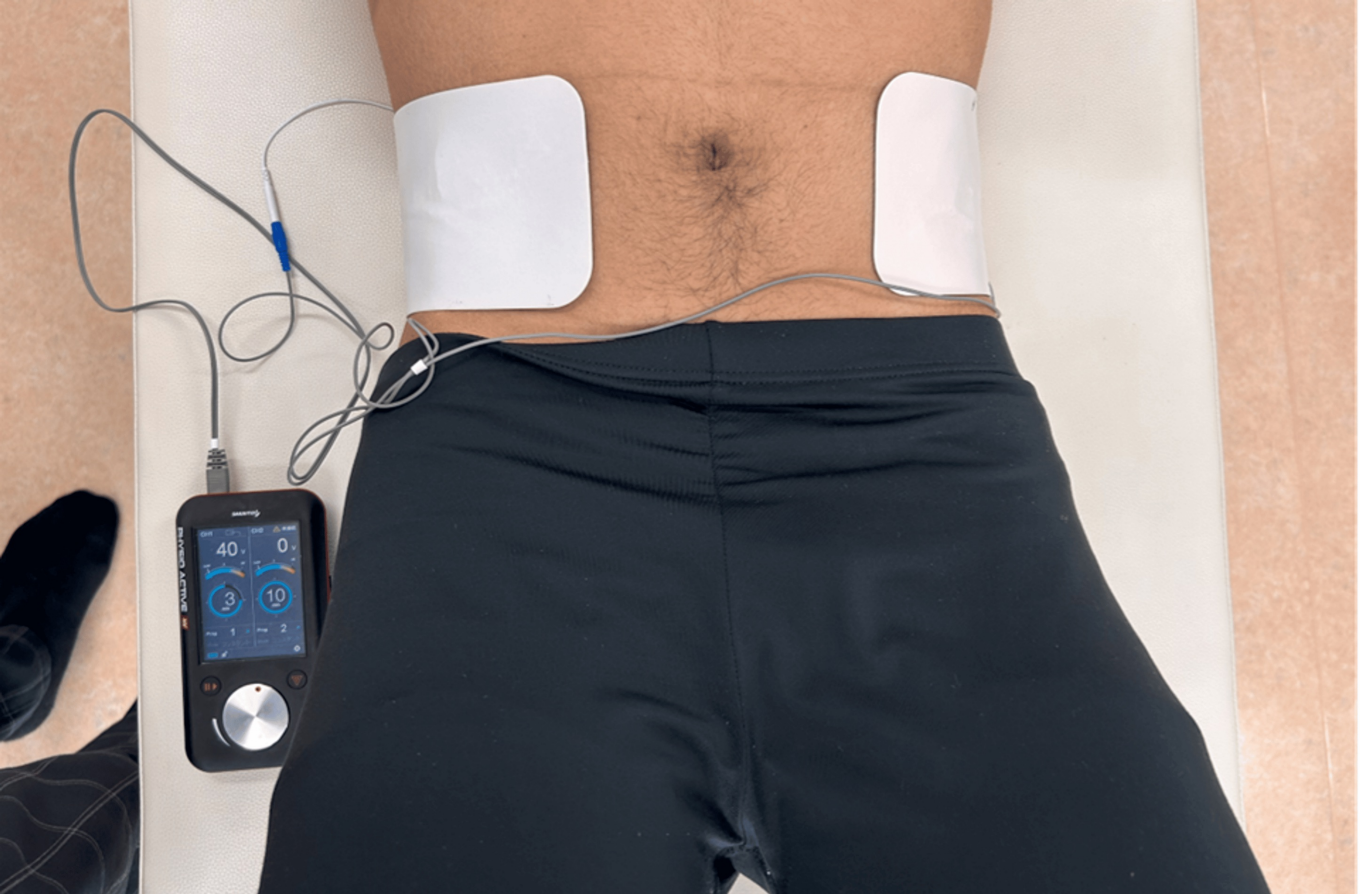
Electrode pads attached to the abdomen in the HVPC group during the hollowing exercise HVPC, high-voltage pulsed-current

First, the participants were provided an explanation of the experimental procedure. The HVPC group simultaneously performed abdominal hollowing exercises for 4 minutes. The participants were placed on a bed in a supine position with their knees flexed and feet flat. An HVPC device (PHYSIO ACTIVE HV; SAKAI Medical Co. Ltd., Tokyo, Japan) was used to contract the transversus abdominis muscle in the intervention group. Electrode pads (100 mm × 198 mm) were attached to the bilateral abdomen between the iliac crest and the 12th rib. The stimulation parameters comprised a frequency of 30 Hz, pulse duration of 50 µs, and on/off ratio of 3/3 s. Before the experiment, we virtually checked the contraction of the transversus abdominis using ultrasound, and this protocol was then decided. The electrical stimulation intensity was adjusted to reach the maximum tolerable intensity without discomfort. The average intensity was 36.5 ± 5.2 V (range 30-50 V) (Figure 1). The abdominal hollowing exercise was performed while electricity flowed. We selected this exercise because it is used for core muscle training [20]. This exercise was performed verbally using the method described by Lee et al. [18]. Throughout the intervention, participants were monitored for adverse responses, including excessive discomfort, pain, or muscle fatigue. The procedure was immediately terminated if adverse reactions were observed or reported by the participants.

The control group was fitted with electrode pads but did not receive an electric current and performed abdominal hollowing exercises for 4 minutes.

Measurements

**Figure 2:**
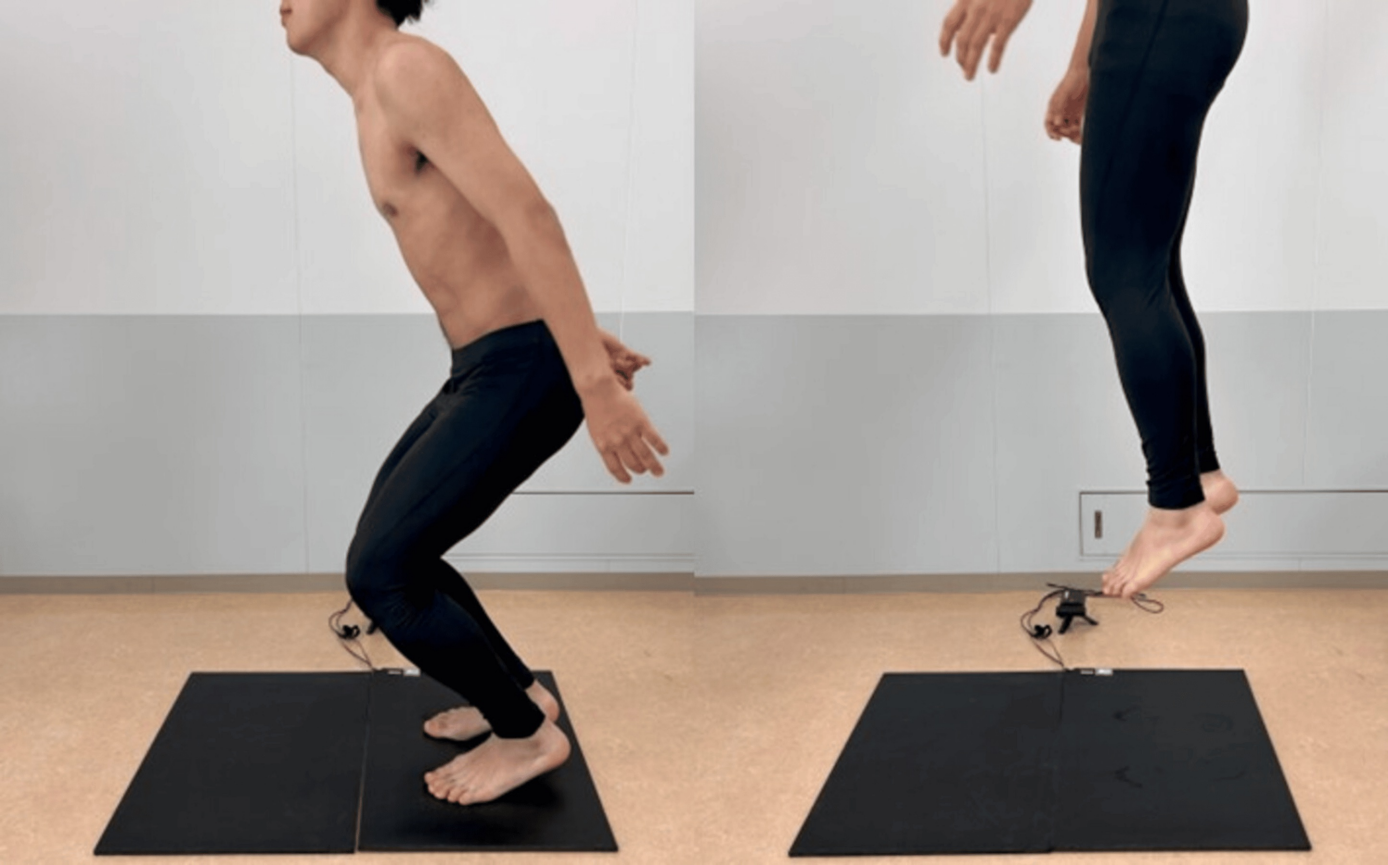
Experimental setup of the rebound jump test Participants performed six consecutive vertical jumps on a jump mat while minimizing the ground contact time.

Rebound jump (RJ) index: The participants performed six consecutive vertical jumps (Figure 2), as described in a previous study [21,22]. They were instructed to jump as high as possible with the shortest possible ground contact time while being free to use their arms during the jump execution. The RJ index was measured using a jump mat (S-CADE Co., Tokyo, Japan). No instructions were provided during the performance to avoid bias. The first of the six trial jumps was not evaluated. Jump height, ground contact time, and the RJ index for each jump were measured using Volt ono jump (S-CADE Co., Tokyo, Japan). The RJ index was calculated by dividing the jump height by the ground contact time. The participants practiced RJ for one week leading to the day of the experiment, and we confirmed that the exercise was correctly executed.

Statistical analysis

The data are expressed as mean ± standard deviation. Normality was evaluated with the Shapiro-Wilk test, and baseline characteristics between groups were compared using unpaired t-tests. Paired t-tests were used to compare the pre- and post-experiment jump heights, ground contact time, and RJ index. The level of significance was set at p < 0.05. All statistical analyses were performed using IBM SPSS Statistics for Windows (version 27.0; IBM Corp., Tokyo, Japan).

## Results

There were no significant differences in demographic information between the HVPC and control groups (Table 1).

**Table 1:** Baseline characteristics of the HVPC and control groups Data are presented as mean ± standard deviation. BMI, body mass index; HVPC, high-voltage pulsed-current

Variable	Age (years)	Height (cm)	Weight (kg)	BMI
HVPC group (n = 18)	20.5 ± 1.4	167.5 ± 5.0	58.4 ± 7.8	20.7 ± 1.9
Control group (n = 18)	20.6 ± 1.1	167.1 ± 8.1	59.3 ± 6.7	21.2 ± 1.2
Total (n = 36)	20.6 ± 1.3	167.3 ± 6.6	58.8 ± 7.2	20.9 ± 1.6
p-value	0.81	0.79	0.72	0.97

Table 2 presents the changes in jump height, ground contact time, and RJ-index pre- and post-intervention for the HVPC and control groups. The HVPC group showed a significant increase in jump height and the RJ index after the intervention but no change in ground contact time. The control group showed no significant differences in any of the items before and after the intervention.

**Table 2:** Comparison of pre- and post-intervention outcomes Data are presented as mean ± standard deviation. *Significant difference compared to the pre-intervention values. HVPC, high-voltage pulsed-current; RJ index, rebound jump index

Variable	HVPC group			Control group			
	Pre	Post	p-Value	Pre	Post	p-Value	
Jump height (cm)	31.95 ± 0.38	33.64 ± 0.31*	0.01	32.10 ± 0.33	32.40 ± 0.36	0.83	
							Contact time (s)	0.20 ± 0.07	0.20 ± 0.06	0.89	0.20 ± 0.05	0.20 ± 0.05	0.8		
RJ index	1.67 ± 0.03	1.75 ± 0.03*	0.02	1.65 ± 0.03	1.68 ± 0.03	0.64									

## Discussion

This study examined the immediate effects of short-duration exercise with HVPC stimulation on the trunk muscle performance. The results of this study showed that 4 minutes of exercise with HVPC stimulation improved jump height and the RJ index. Warm-ups before a competition need to improve performance without inducing fatigue in a short period. The results of this study suggest that passive warm-ups with the transversus abdominis muscle being contracted using HVPC stimulation may benefit athletes without causing fatigue.

In the HVPC group, the intervention increased the jump height by 1.7 cm and improved the RJ index by 0.08. However, there was no change in contact time. This indicates that the intervention may have resulted in a greater power gain within the same contact time. In Fletcher's study, RJ was 1.3 cm after a 10-minute general warm-up, and in our previous study, it was 1.6 cm after a 4-minute active warm-up using running. Therefore, we hypothesize that a 4-minute HVPC stimulation session would have the same effect as a general warm-up [22,23]. The warm-up strategy used in this study may be particularly suitable for sports such as volleyball and basketball, where jumping as high as possible is necessary.

Conversely, no changes were noted in the control group after the intervention. Previous studies have reported that trunk muscle training, including abdominal hollowing exercises, contributes to improved functional performance and injury prevention in a wide range of age groups [24,25]. Additionally, a recent review of core training in athletes reported its effects on balance performance, throwing distance, jump performance, and running economy [26,27]. Therefore, trunk muscle training is widely used as a warm-up [28,29]. Particularly, deep core muscles such as the transversus abdominis and multifidus are activated before limb movements, such as the active straight leg raise, contributing to improved movement efficiency [30]. However, the abdominal hollowing exercises alone did not affect performance in the control group. According to Lee et al., the contraction rate of the transversus abdominis muscle was higher when ultrasonography-based feedback was provided compared with verbal instructions alone [18]. Specifically, it may be challenging to induce maximal contraction through voluntary contraction using only verbal instructions. Therefore, participants in the control group may not have warmed up sufficiently. Given that contraction of the transversus abdominis muscle can be easily evaluated using ultrasonography, future research should also investigate the extent of change in muscle thickness to ensure comparable contractility across participant groups.

This study has some limitations. First, our study only measured jump performance, and possible effects on other physical performance metrics and biological mechanisms remain unknown. Warm-ups generally enhance muscle metabolism, fiber conduction velocity, contractile performance following prior contractile activity, and VO2 kinetics [4]. Considering that this study involved direct stimulation, its impact on respiratory function was likely minimal. Therefore, HVPC-based passive warm-ups should not be viewed as a complete replacement but rather as a complementary method. Second, we did not measure the exact contraction of the transversus abdominis muscle during the intervention. Future studies should evaluate changes in transversus abdominis muscle thickness using ultrasound to better understand its activation and contribution to performance outcomes. Third, the protocol in this study was set to 4 minutes to allow for comparison with previous studies; however, whether this stimulation duration was optimal remains unclear. Future research should explore different stimulation durations to develop a more practical and effective protocol for sports settings. Fourth, Fletcher [23] demonstrated that combining general warm-up, dynamic stretching, and squatting increased jump height by 5.5 to 7.0 cm in collegiate athletes. Therefore, integrating HVPC stimulation into other warm-up methods may lead to more effective strategies. Finally, this study only evaluated immediate effects, and its long-term effects remain unclear. Given that warm-up benefits should last at least 10-20 minutes [28], future studies should examine the duration of HVPC stimulation’s effects.

## Conclusions

We examined the effects of trunk muscle training with HVPC stimulation on jump performance. In healthy young participants, jump performance improved when HVPC stimulation was applied during 4-minute trunk muscle training. This approach may enable a quicker warm-up compared to an active warm-up without causing energy depletion. Therefore, HVPC-assisted warm-ups show potential, particularly in sports.
